# Influence of an allogenic collagen scaffold on implant sites with thin supracrestal tissue height: a randomized clinical trial

**DOI:** 10.1007/s00784-024-05716-0

**Published:** 2024-05-15

**Authors:** A Solderer, SP Hicklin, M Aßenmacher, A Ender, PR Schmidlin

**Affiliations:** 1 Clinic of Conservative and Preventive Dentistry, Center of Dental Medicine, University of Zurich, Zurich, Switzerland; 2 Department of Statistics, LMU Munich, Munich, Germany; 3 Munich Center for Machine Learning (MCML), LMU Munich, Munich, Germany

**Keywords:** Soft-tissue height, Marginal bone level, Bone remodeling, Dental implant, Collagen scaffold, Acellular dermal matrix

## Abstract

**Objectives:**

This randomized clinical trial focused on patients with thin peri-implant soft-tissue height (STH) (≤ 2.5 mm) and investigated the impact of an allogenic collagen scaffold (aCS) on supracrestal tissue height and marginal bone loss (MBL).

**Material & methods:**

Forty patients received bone level implants and were randomly assigned to the test group with simultaneous tissue thickening with aCS or the control group. After three months, prosthetic restoration occurred. STH measurements were taken at baseline (T0) and reopening surgery (TR), with MBL assessed at 12 months (T1). Descriptive statistics were calculated for continuous variables, and counts for categorical variables (significance level, *p* = 0.05).

**Results:**

At T1, 37 patients were available. At T0, control and test groups had mean STH values of 2.3 ± 0.3 mm and 2.1 ± 0.4 mm. TR revealed mean STH values of 2.3 ± 0.2 mm (control) and 2.6 ± 0.7 mm (test), with a significant tissue thickening of 0.5 ± 0.6 mm in the test group (*p* < 0.03). At T1, control and test groups showed MBL mean values of 1.1 ± 0.8 mm and 1.0 ± 0.6 mm, with a moderate but significant correlation with STH thickening (-0.34), implant position (0.43), history of periodontitis (0.39), and smoking status (0.27).

**Conclusion:**

The use of an aCS protocol resulted in soft tissue thickening but did not reach a threshold to reliably reduce MBL compared to the control group within the study’s limitations.

**Clinical relevance:**

Peri-implant STH is crucial for maintaining peri-implant marginal bone stability. Marginal bone stability represents a crucial factor in prevention of peri-implantitis development.

**German register of clinical trial registration number** DRKS00033290.

## Introduction

The establishment and maintenance of adequate peri-implant soft and hard tissue conditions are crucial for ensuring the long-term success of dental implants [1]. In addition to a sufficient bone bed, often referred to as the “bony envelope” [2], soft tissue integration remains essential [1]. Soft-tissue integration is a complex biological process influenced by various factors [3]. A key element of this biological equilibrium is the supracrestal tissue height (STH) [4], comprising primarily an epithelial and a connective tissue segment, playing a central role in the remodeling of peri-implant tissues post-implant surgery and prosthetic loading. Under healthy physiological conditions, STH is histologically characterized by an approximate 2 mm epithelial barrier coupled with a connective tissue component spanning 1–2 mm, resulting in an overall height of 3–4 mm. In this context, short and long supra-crestal soft tissue heights (STH) with a cut-off of 3 mm thickness have been described [4]. Any deviation from this equilibrium, particularly when the mucosa around implants becomes rather thin (≤ 2 mm), has been associated with early marginal remodeling and bone loss after abutment connection. This ultimately leads to the establishment of a “minimal” peri-implant STH [5, 6].

In 1996, Abrahamsson and colleagues were the first to demonstrate that sites with thin crestal mucosa around implants (≤ 2 mm) exhibit marginal bone loss, resulting in remodeling and the establishment of a “minimum” of supracrestal tissue height (STH), similar to regular or thick biotypes [7]. They concluded that a specific height of peri-implant mucosa was necessary to ensure a proper epithelial-connective tissue attachment apparatus. Subsequent animal studies supported this concept and affirmed a minimum mucosa height of at least 2 mm for the establishment of a stable soft tissue seal [8]. Consequently, criteria for physiological bone loss during the first year of loading and beyond were established for machined surface implants and two-stage surgical procedures, reflecting initial peri-implant bone remodeling as a consequence of the biologic adaptation of peri-implant tissues until a steady-state condition can be expected [9–11]. A meta-analysis of three different implant systems revealed a mean bone loss of 1 mm within the first five years after loading, with the majority occurring in the initial year [12]. In a more recent retrospective study 0.5 mm of marginal bone loss six months after implant loading emerged as a suggested benchmark for success [13].

The research group led by Linkevicius initially analyzed bone remodeling in patients with specifically thin supracrestal tissue height (STH) (≤ 2 mm), utilizing platform-matching and platform-switching implants [5, 14]. They found no statistical difference between the groups but identified an overall marginal bone loss of up to 1.8 mm after one year [5, 14]. Subsequently, bone remodeling and resorption in patients with varying STH were determined, confirming that STH under 2 mm exhibited the largest bone resorption, averaging 1.2 mm, while patients with greater thickness showed inversely lower values, down to zero for an STH of 4 mm [15]. Based on these findings, a minimal threshold of 3 mm could be established. A meta-analysis supported this assumption, demonstrating that implants placed in initially thicker peri-implant soft tissues exhibit less radiographic bone loss, at least in the short term [16]. Therefore, a targeted conceptual thickening of thin mucosa can lead to less crestal bone loss [6]. Not surprisingly, a recent consensus study emphatically stated, “bone stands hard, but soft tissue is the guard,” highlighting the bidirectional importance of hard and soft tissue interrelationships [17]. Since then, various approaches for tissue thickening have been described, employing a plethora of graft materials [6, 18, 19]. Particularly, alternatives to autogenous grafts have spurred the development and evaluation of various matrices of either allogenic or xenogeneic origin [19]. This addresses the need for an additional surgical site while offering an unlimited supply [17] and potentially reducing surgical time [18]. In this clinical study, the primary aim of this study was to evaluate the effectiveness of an allogenic dermal matrix (aCS) in increasing peri-implant soft tissue height (STH) compared to no additional tissue modification in sites with an initially thin phenotype (≤ 2.5 mm) when placed over the bone crest simultaneously with implant placement. As a secondary objective, the extent of bone remodeling was assessed. The authors hypothesized that the application of a soft-tissue matrix material post-healing would significantly contribute to mucosal thickening. The secondary research hypothesis posited that bone resorption or remodeling, particularly marginal bone loss, within the test group would be notably less pronounced compared to the control group after a 12-month period.

## Material & methods

### Study design

This trial was designed as a single-center, parallel-group, randomized, controlled trial. The study was conducted in compliance with the investigation plan, the current version of the Declaration of Helsinki, ISO EN 14,155, as well as national legal and regulatory requirements. The protocol received approval from the responsible authorities (ID: 2020-00037) and was registered in the German register of clinical studies (DRKS00033290). The study was carried out at the University of Zurich. CONSORT guidelines were adhered to as per protocol [20].

### Study population

Forty patients were recruited, with 20 patients assigned to each treatment group. Inclusion criteria encompassed individuals requiring a single implant in the posterior area, specifically premolars and molars in the maxilla or mandible, intended for a single-tooth implant-supported crown. Additional inclusion criteria were as follows:

- Systemically healthy.
- Smoking ≤ 10 cig./day.
- No horizontal bone augmentation required.
- Mucosa thickness ≤ 2.5 mm.

Patients were excluded if they presented any of the following conditions: Heavy smoking (> 10 cig./day); poor oral hygiene after the hygienic phase; active periodontal disease (residual pockets > 4 mm); the need for horizontal bone augmentations; sites with a previous ridge preservation, a history of radiation in the head-neck area; systemic or local diseases or conditions that could compromise healing or osseointegration; use of drugs influencing bone metabolism; severe bruxism; and poor compliance or unwillingness to return for follow-ups.

### Randomization, allocation concealment and blinding

Due to the exploratory nature of this low-scale and pilot study, the sample size calculation relied on rough assumptions and considerations of relevant differences between the groups. Including 18 patients per group, a Wilcoxon rank-sum test was estimated to have 80% power to detect a difference of 1 mm in mucosa change between the two groups, with a significance level α = 0.05 and assuming a standard deviation of 1 mm in each group. Accounting for a drop-out rate of approximately 10%, a final inclusion of 20 patients per group was determined, totaling 40 participants.The randomization between positions for either test or control implants was carried out using sealed envelopes by the trial statistician. Clinical and radiographic measures, as well as statistical analyses, were conducted in a blinded manner with respect to treatment assignment. All radiographic measurements were performed by two experienced researchers (P.R.S & S.P.H) who were blinded to the treatment groups.

### Study interventions

- All patients underwent a hygiene phase, which included information about plaque-induced diseases, instruction, and motivation for oral hygiene measures, along with professional tooth cleaning. Inclusion in the study was contingent upon patients meeting the following parameters: Full-mouth plaque Index ≤ 30%.
- Full-mouth bleeding on Probing ≤ 30%.
- Probing depth at all teeth ≤ 4 mm.

**Fig. 1:**
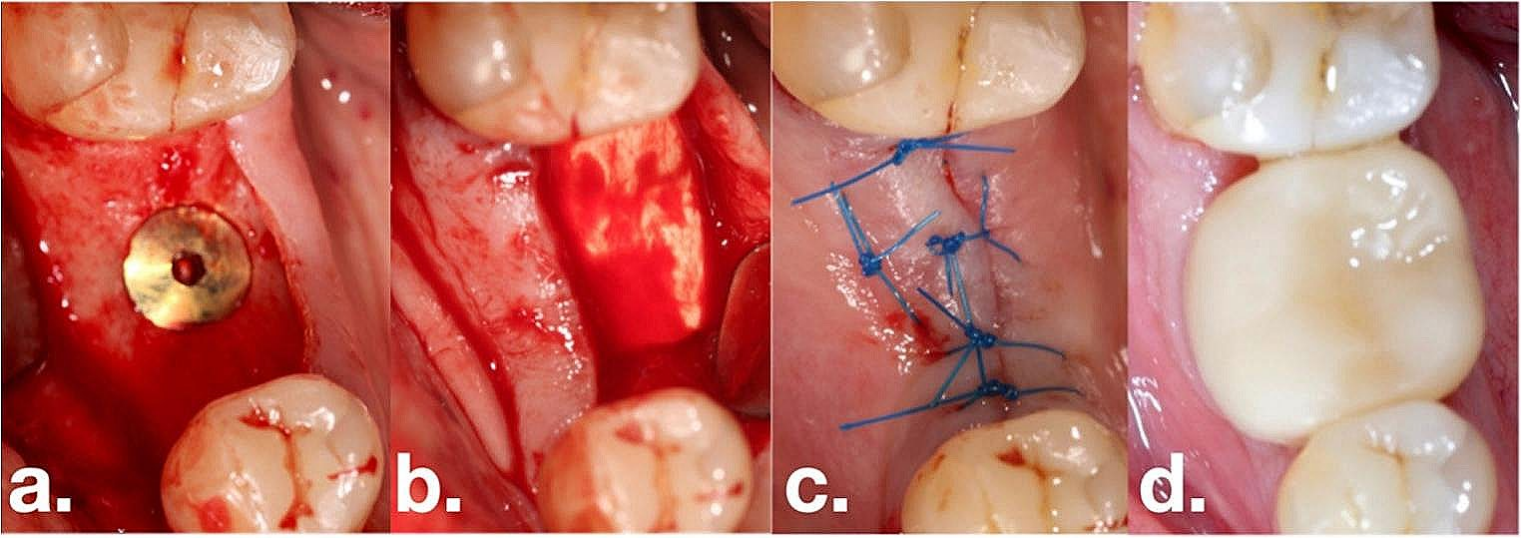
Test-group patient: **(a)** Bone-level implant placed at bone crest (T0), **(b)** Collagen scaffold in position. **(c)** Abutment connection at TR. **(d)** 12-months follow-up (T1) with crown in place

All surgeries (Fig. 1) were performed by two experienced specialists and surgeons (A.S. & S.P.H). After local anesthesia, a local flap surgery was conducted to appropriately place a bone-level implant. During the implant installation, (T3 Tapered, non-platform switch, ZimVie Dental), before flap closure, the periosteum of the buccal flap was released for all implants to allow for tension-free advancement and suturing of the flaps. Implants were placed exactly at the level all rough surfaces were covered by bone, seeking for an epicrestal positioning of the implant shoulder. Patients were then randomly assigned to the control and test groups. The latter group received an allogenic collagen scaffold (Tutogen Medical GmbH, Puros Dermis Allograft Matrix 0.8–1.8 mm), which covered the implant cover screw and the crestal part of the surrounding bone; it was secured by placing a small part of the matrix under the buccal and oral flap. All implants were then subjected to submerged healing. Suturing was performed using routine suture techniques, depending on the clinical situation, to achieve primary closure (i.e., single, double mattress, and/or double loop sutures). In the control group, no grafting material was applied. However, the periosteum was also released to ensure comparable tissue manipulations and conditions. After surgery, the patient was instructed to maintain adequate oral hygiene at the surgical site and to rinse twice a day with a 0.12% chlorhexidine solution until sutures were removed 7–10 days after surgery. After 10–12 weeks, abutment connection was performed (BellaTek Encode 2-piece healing abutment, ZimVie Dental). Approximately two weeks after this uncovering surgery, the optical scans were performed with Cerec Primescan (DentsplySirona, Bensheim, Germany) and monolithic full ceramic zirconia single crown (Katana Zirconia YML, Kuraray Noritake, Dental Inc., Tokyo, Japan) using Exocad Lab-Software (Exocad 2.2, Darmstadt, Germany) were fabricated using an lab milling device (inLab MC X5, DentsplySirona) in combination with non-platform switching Zfx GenTek TiBase, Certain Flex abutment (ZimVie Dental).

### Clinical & radiographic examinations

#### Assessment of the primary outcome

**Fig. 2:**
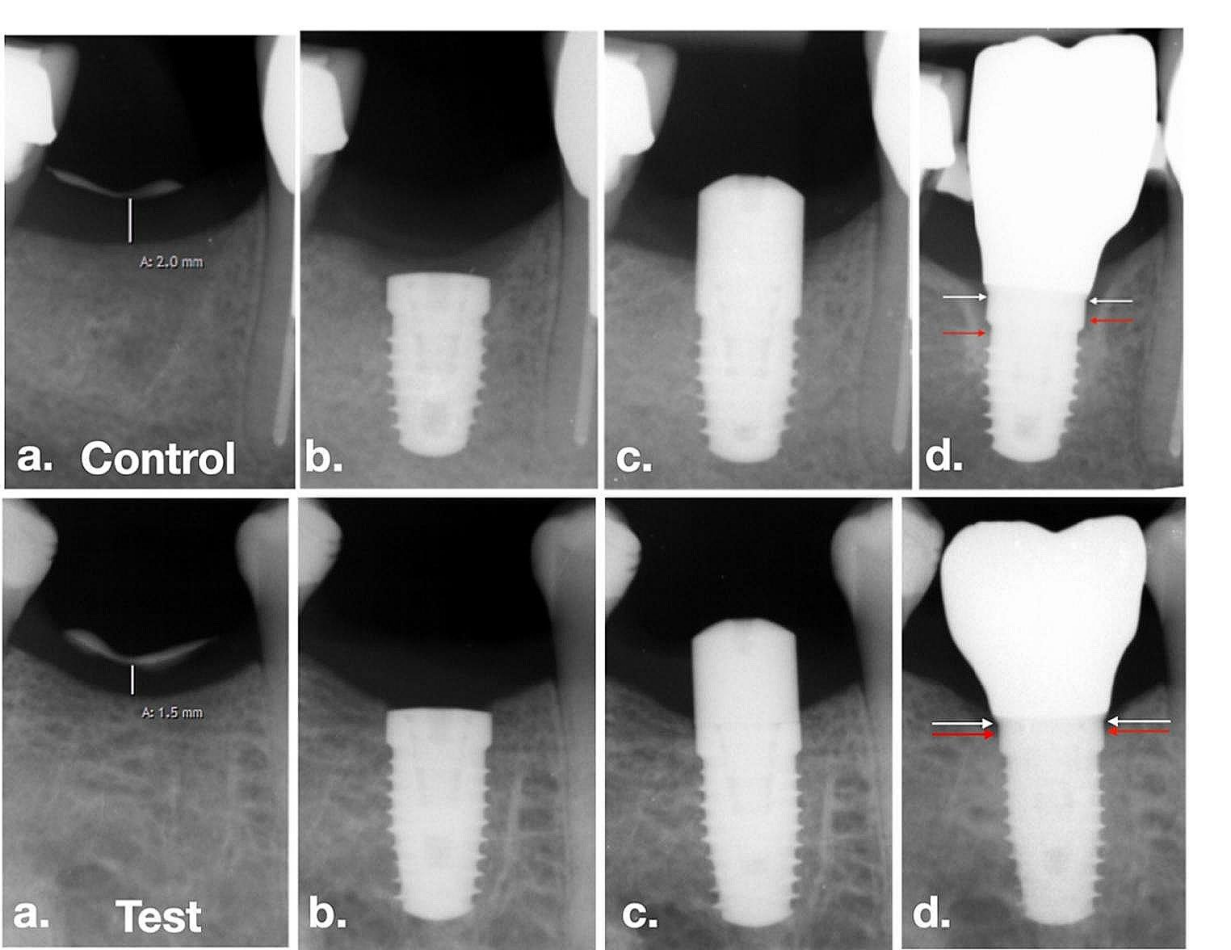
Test-group and control group patient examples: **a**. Preoperative radiographic measurement for patient inclusion **b**. Bone-level implant placed at bone crest (T0) **c**. Abutment connection at uncovering surgery (TR) **d**. 12-months follow-up (T1) with marked implant shoulder (white arrows) and marginal bone level (red arrows)

Supracrestal tissue height (STH) was assessed through single X-rays, with a flowable composite line serving as a marker on the top of the crestal mucosa for patient inclusion (Fig. 2a). During implant and reopening surgery, flap thickness was measured using a calibrated periodontal probe (PCP-UNC 15 Hu-Friedy, Chicago, IL, USA), rounding to the closest 0.5 mm. Measurements were always done in the projected future implant position and carried out by the team of the same two surgeons (A.S. & S.P.H.).

#### Assessment of secondary outcomes

Standardized x-rays at implant sites were taken using a paralleling technique with Rinn holders and analogue films (Kodak Ektaspeed Plus, Eastman Kodak and Co., Rochester, NY, USA) at visits 1, 3, 5, and 7. The films were digitized, calibrated using the diameter of the implant and the marginal bone level, and assessed at a 10x to 15x magnification using open-source software (Image J; National Institutes of Health, Bethesda, MD, USA). The distance between the implant shoulder and the bone crest was measured at the mesial and distal aspects of the implants to the nearest 0.01 mm (distance implant shoulder to bone = DIB). Marginal bone level changes were assessed between the different time points T0 and T1 (Fig. 2b-d).Pocket probing depths (PPD) and bleeding on probing (BOP), assessed via a periodontal probe (PCP-UNC 15 Hu-Friedy, Chicago, IL, USA), were recorded at T1, 12 months after crown delivery.

### Statistical analysis

Sample size calculation was conducted under cautious and conservative assumptions. A Wilcoxon rank-sum test, not relying on the normality assumption of the data, was employed to calculate the sample size needed to detect a relevant difference in mucosa change of 1 mm between the treatment and control groups. Requiring the test to have a power of 80% and a significance level of 5% resulted in the inclusion of 20 patients per group, accounting for potential dropouts, and a total of 40 patients. Excel (version 16.70, Microsoft, Redmond, Washington, USA) was utilized for coding and documenting the data, while the statistical software package R (R Core Team, 2021) [21] was employed for the analysis. Descriptive statistical measures included the arithmetic mean alongside standard deviations, the median, and several quantiles, which were computed for creating boxplots. Primary and secondary endpoints were formally analyzed using statistical tests. The authors conducted an analysis of variance (ANOVA), with the treatment indicator as the influential variable of interest. Additionally, the model was controlled for three confounding factors (smoker status, history of periodontal treatment, region of the implant). Due to extremely small sample sizes in all subgroups for different covariate combinations, it was not possible to formally test the normality assumption. However, given the nature of the target variable (continuous, differences between two time points), it is reasonable to assume normality. To determine the significance of the differences between the treatment and control groups, a significance level of 0.05 was employed. The primary research hypothesis postulates that employing the treatment (a soft-tissue matrix material after healing) significantly thickens the mucosa.The secondary research hypothesis posits that bone resorption or remodeling, specifically marginal bone loss (MBL), in the test group is less pronounced than in the control group after 12 months. Additionally, pairwise Pearson correlation coefficients were calculated between MBL and (i) the difference in mucosa thickness, (ii) smoker status, and (iii) history of periodontal treatment. These correlation measures were complemented by calculating Odds Ratios for the association between a binarized variant of MBL (> 1 mm yes/no) and (i) smoker status and (ii) history of periodontal treatment. Considering that the region of the jaw is measured on a nominal scale, the association between MBL and the region of the jaw was assessed using Cramer’s V as a correlation measure.

## Results

### Study population

**Fig. 3:**
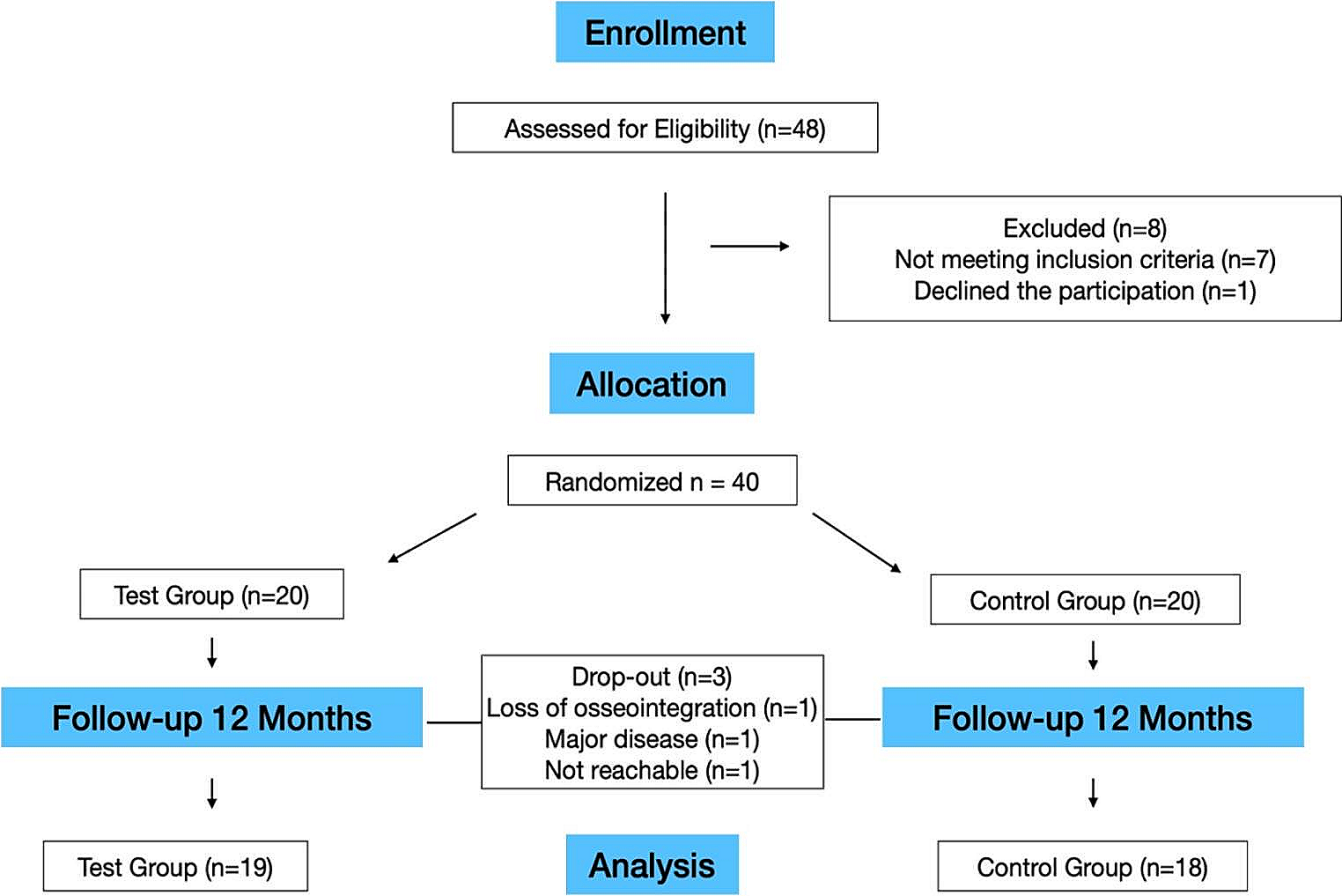
Consort flow-diagram

Patient recruitment took place between July 2020 and May 2022. As per the initial plan, forty patients were enrolled, all of whom received their final restorations. Six patients underwent simultaneous sinus grafting procedures. However, three patients did not complete the 12-month follow-up (2 in the control group, 1 in the test group). In the test group, one implant was lost six months after crown delivery, possibly due to overloading (any data from this patient were included). Additionally, one patient became unreachable, and another patient underwent the extraction of all teeth and implants for oncologic reasons *alio loco*. Figure 3 provides an overview of the enrollment and allocation process, while Table 1 describes the baseline assessments.

**Table 1:** General characteristics of the included patients

Patient-level characteristics	Overall (*n* = 37)	Control (*n* = 18)	Test (*n* = 19)
Age (years), mean (SD)		57.9 (13.5)	56.5 (15.2)
**Gender N (%)**			
Male	21 (57%)	11	10
Female	16 (43%)	7	9
**Smoking status N**			
Non-smoker	27	13	14
Light Smoker (< 10 cig./d)	10	5	5
**History of Periodontal Disease N**			
Yes	14	6	8
No	23	12	11
**Soft-tissue Height at T0**			
**Mean (SD)**		2.28 (0.26)	2.13 (0.44)
**Implant position N**			
Upper Jaw	19	10	9
Lower Jaw	18	8	10

### Clinical outcomes

An overview of the clinical outcomes at T0 and T1 is provided in Table 2.

**Table 2:** Clinical and radiographic outcome measures for control and test group

Soft tissue height			
T0 - Baseline	Control	*p*- value	Test
Mean ± SD	2.28 ± 0.26		2.13 ± 0.44
**TR (Re- Opening)**			
Mean ± SD	2.33 ± 0.24		2.63 ± 0.66
**Change**			
Mean ± SD	0.06 ± 0.16	0.003	0.50 ± 0.62
**Marginal Bone Loss**			
**T1**	**Control**	**p- value**	**Test**
Mean ± SD	1.06 ± 0.80	0.60	0.96 ± 0.56
**Pocket Probing Depth**			
**T1**	**Control**		**Test**
Mean ± SD	3.0 ± 0.6 mm		3.3 ± 1.0 mm
**Bleeding on Probing**			
**T1**	**Control**		**Test**
Mean ± SD	25.9 ± 30.9%		34.2 ± 30.2%
**Keratinized Tissue (buccal)**			
**T1**	**Control**		**Test**
Mean ± SD	2.9 ± 1.0 mm		3.0 ± 1.4 mm

#### Primary clinical outcome - soft tissue height

Soft tissue height (STH) at baseline (T0) revealed no significant difference, measuring 2.3 ± 0.3 mm in the control group and 2.1 ± 0.4 mm in the test group. Upon reopening (TR), the control group demonstrated an overall unchanged thickness of 2.3 ± 0.2 mm, while the test group exhibited a slightly increased thickness of 2.6 ± 0.4 mm. Consequently, a statistically significant (*p* < 0.33) thickening of the soft tissue was observed, with a mean increase of 0.5 ± 0.6 mm compared to the control group. In contrast, the control group displayed nearly the same thickness (an increase of 0.06 ± 0.16 mm) compared to T0 (Fig. 1).

#### Secondary clinical outcomes

Regarding buccal KT width at T1, no statistical significance was observed. The control and test groups exhibited respective means of 2.9 ± 1.0 mm and 3.0 ± 1.4 mm. Additionally, Probing Pocket Depth (PPD) at T1 was recorded at 3.0 ± 0.6 mm in the control group and 3.3 ± 1.0 mm in the test group. Bleeding on Probing (BOP) at T1 revealed that 25.9 ± 30.9% of implants in the control group exhibited at least one measuring point with BOP, whereas the test group displayed BOP in 34.2 ± 30.2% of the implants.

### Radiographic outcomes

#### Marginal bone loss

**Fig. 4:**
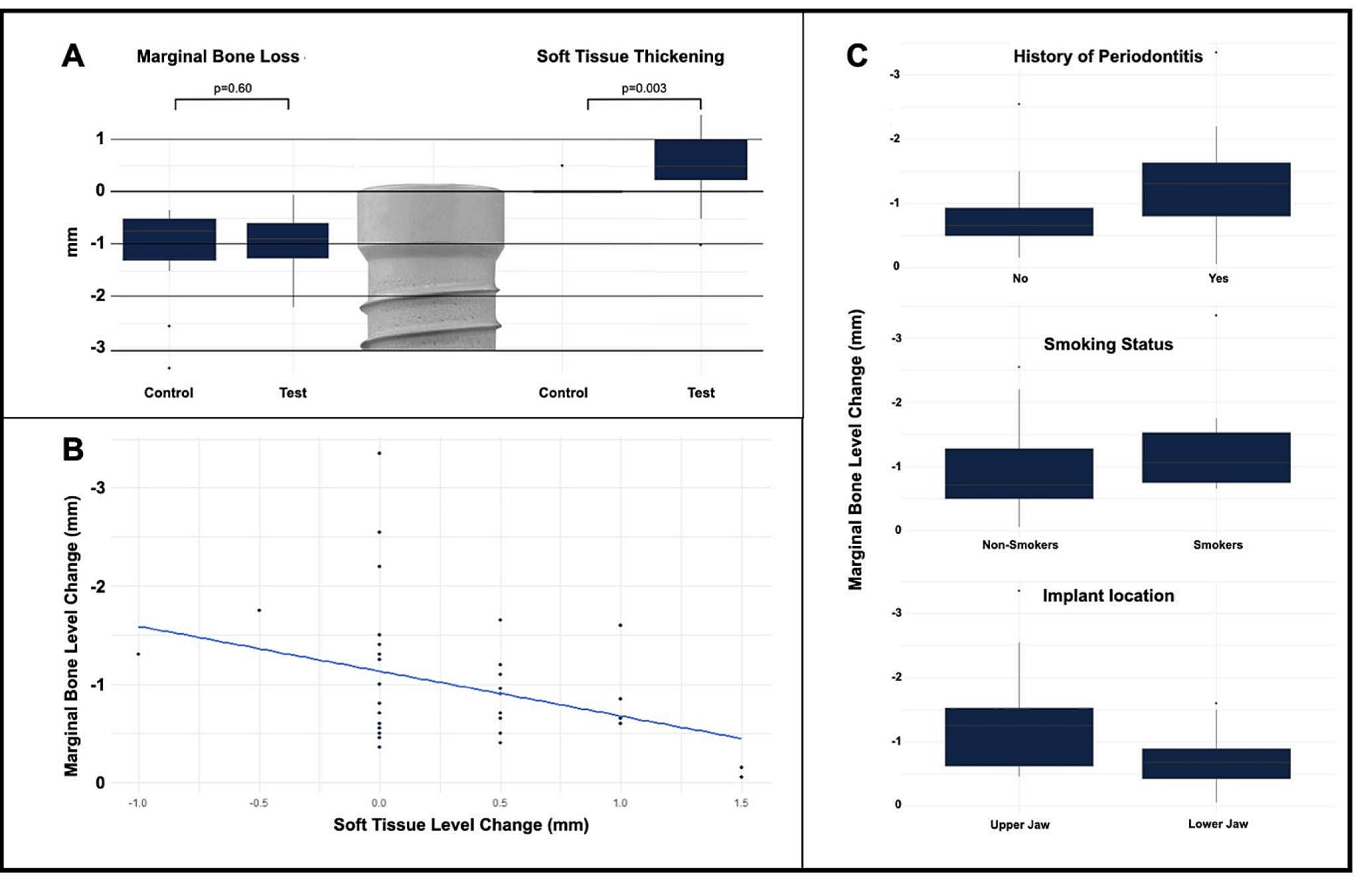
Box-plots and correlation for **A** & **B**: MBL (mm) and STH thickening (mm), **C**: Correlations for MBL and History of Periodontitis, Smoking Status and Implant Region (right)

At T1, the control group demonstrated an overall mean marginal bone loss (MBL) of 1.1 ± 0.8 mm, whereas the test group exhibited a slightly lower MBL of 1.0 ± 0.6 mm. The disparity between the two groups was determined to be statistically insignificant (*p* > 0.5). Notably, a negative correlation (-0.34) emerged between soft tissue height (STH) and MBL. This correlation signifies a reduction in MBL for patients with thicker STH compared to those with a thinner one (Fig. 4). Significantly divergent trends were also observed between implants placed in the upper and lower jaws, as well as among patients with a history of periodontitis and those classified as light smokers (< 10 cig. / day) versus non-smokers (Fig. 4). Specifically, implants in the upper jaw manifested a notably greater degree of bone loss (*p* > 0.02) when compared to those in the lower jaw among all included patients. Cramer’s V measure was computed, yielding a value of 0.4249, indicating a medium to high association between the two variables. Patients with a history of periodontitis (*n* = 14) demonstrated a substantial correlation (0.39) with higher MBL in both treatment groups. The calculated odds ratio (OR) revealed an 11.9 times higher risk for patients with a history of periodontitis to develop MBL over 1 mm, as opposed to patients without a history of periodontitis (*n* = 23). A notable difference (*p* > 0.06) was observed between light smokers (*n* = 10) and non-smokers (*n* = 27) in terms of developing higher MBL. Light smokers exhibited twice the risk (Odds Ratio = 2) for MBL exceeding 1 mm.

## Discussion

The clinical significance of this research lies in the potential to address undesirable bone resorption and remodeling following implant placement and abutment connection. Bone remodeling can lead to the exposure of implant microrough threads, resulting in plaque accumulation in difficult-to-clean sites and subsequent peri-implant inflammation [20]. Presently, peri-implantitis remains a primary cause of implant failure [21]. Ravida´ and colleagues [22] delved into the role of exposed implant threads after initial bone remodeling, emphasizing an eight-fold greater odds ratio (OR) compared to non-exposed implants for the development of peri-implantitis. Importantly, the risk increases four-fold with each additional exposed thread. The authors thus concluded that exposed interproximal implant threads following physiological bone remodeling may serve as an independent risk indicator for the development of peri-implantitis. In this investigation, the authors observed an average bone loss of approximately 1 mm within the first year, consistent with findings from comparable studies such as Canullo’s report indicating a 1.49 mm loss in the first year [23]. While not reaching statistical significance, the test group exhibited a discernible trend toward lesser bone loss (0.96 mm) compared to the control group (1.06 mm). The observed soft tissue thickening in our cohort accounted for 0.5 mm, which may have been insufficient to reliably and completely prevent associated bone loss. In contrast, other studies consistently reported higher gains in soft tissue height (STH). For instance, in a similar study comparing, a STH gain of 2.2 mm was observed in the allogenic acellular dermal matrix (ADM) group. It is important to note that the same material was used as in our study; however, the other study employed a double-layer technique to achieve a thickness of 2–3 mm, as described by the authors [6]. Unlike our study, they utilized Straumann bone level (BL) platform-switching implants with a transgingival healing protocol, which may partially explain the differing results. In a subsequent study, the same authors employed a porcine collagen scaffold of 2 mm thickness and successfully thickened the STH from a mean of 1.7 to 3.5 mm, representing an average thickening of 1.8 mm [24]. Another study compared xenogeneic matrices and autogenous CTG in buccal and crestal thickening. Both thickening methods resulted in less MBL compared to no grafting; however, no statistical difference between the intervention groups was found [25, 26]. One clinical study presented conflicting results wherein connective tissue grafts were employed to augment crestal soft-tissue height. Despite a mean increase in soft tissue height of 1.2 mm in the connective tissue graft (CTG) group, the difference was not statistically significant. However, noteworthy was the observation that more bone loss occurred in the grafted implants after 12 months (0.8 mm) compared to the non-grafted implants (0.6 mm) [28]. It is plausible that this discrepancy could be attributed to the relatively small sample size of only 10 patients per group. Recent meta-analyses by summed these results up and found a difference of 0.4–0.5 mm of MBL in the first year between thick and thin STH, favoring the thicker group [27–29]. After three years a smaller non-significant difference of 0.17 mm was found by Tang et al. [28]. An essential consideration in the discourse on effective tissue thickening is the quality of the applied matrix material. In a recent in-vitro trial, the authors observed a low swelling behavior of the allogenic acellular dermal matrix (ADM) used herein, in comparison to a xenogeneic collagen matrix (CM) [30]. This difference was attributed, in part, to the CM’s sponge-like, multilayered structure as opposed to the more compact structure of an ADM. This structural disparity led to the inferior liquid absorption capacity and swelling behavior of the ADM. Another laboratory investigation noted a correlation between larger pore sizes and increased liquid absorption capacities, suggesting a potential superior blood absorption ability [31]. However, it is important to note that a high swelling rate may also contribute to higher rates of wound dehiscences, a common clinical problem that results in faster resorption of exposed connective tissue grafts (CTG) and, consequently, less tissue thickening [32]. Using the herein described protocol, six patients exhibited partial wound dehiscences in the early healing phase. Although these sites showed a tendency toward lower thickening, no statistically significant difference was observed. Moreover, cyclic compression tests assessing the mechanical properties of the ADM revealed minimal elasticity, and the material lost structural integrity with repeated compression. Intriguingly, the native control material also demonstrated minimal compressibility and an inability to sustain its structural integrity. This observation could imply that the allogenic ADM structurally resembles the native material most closely [30]. In addition to soft tissue considerations, various factors such as implant surface characteristics and the design and type of implant connection seem to play a role in influencing peri-implant hard tissue stability. Micro-gaps near the bone can lead to bacterial leakage and may contribute to marginal bone loss [33]. Any inflammatory response related to the implant-abutment interface cannot be entirely compensated for by thicker peri-implant tissues. Studies, albeit conducted in animal models, have suggested that a more apical position of the implant–abutment connection is associated with higher marginal bone loss than a more coronal position. This is attributed to the closer proximity of the inflammation zone to the bone at the implant–abutment interface [34, 35]. Clinically, the use of tissue-level implants with a distinct supracrestal position of the interface region is associated with significantly fewer complications [36]. These findings are supported when comparing bone- and tissue-level implants in patients with thin soft tissue height (< 2 mm) [37]. To counteract the inevitable consequences of bone adaptive processes, particularly as an alternative to soft-tissue thickening, some authors have proposed a subcrestal placement of the implant shoulder by 1–2 mm to minimize initial bone loss [36, 38]. In the herein described protocol, the implants were intentionally placed in an epicrestal manner to ensure that all rough surfaces were completely covered by bone. They featured an internal, almost straight, one-degree conical friction-fit connection [39]. This type of connection exhibits a tight fit concerning angular rotation, pull force, and micromotion of the abutment compared to other conical connections available in the market [39]. Further in-vitro studies have supported these findings, confirming a resilient leakage behavior compared to other abutment connections [40]. Importantly, patient-related factors must also be considered. Correlations between MBL and patient factors demonstrated high significance for implant position, periodontal history, and smoking status in this study. Regarding implant position, there was a higher risk of marginal bone loss for implants placed in the upper jaw compared to those in the lower jaw. An independent analysis also revealed an increase in marginal bone loss in the maxilla of smokers compared to the mandible (standardized mean difference [SMD] 0.40, 95% CI 0.24–0.55; *P* < 0.00001) [41]. In patients with a history of periodontitis, an almost 12-fold higher risk of developing more than 1 mm of MBL was observed. Higher bone loss had previously been documented in individuals with a history of periodontal disease [42]. It is noteworthy that only non-smokers and light smokers (≤ 10 cig./day) were included in this trial. Smokers exhibited a twofold risk of developing MBL exceeding 1 mm compared to non-smokers. A recent radiographic study [43] conducted over at least 36 months also reported more early bone loss in light smokers compared to non-smokers, with the extent of bone loss increasing with the quantity of cigarettes consumed per day. The present study has several limitations, including the relatively small sample size of 20 patients per group and a significant number of patient-related factors, as discussed above. Additionally, it is crucial to consider other material-related and prosthetically driven factors that may impact peri-implant hard and soft tissues. For instance, the transmucosal component can influence the establishment of peri‐implant soft tissue height (STH). A flat and wide emergence profile is known to induce apical displacement of the peri-implant STH, leading to increased bone loss compared to a narrow emergence profile [35]. Restorative angles of less than 40° have recently been highlighted as factors limiting the initial marginal bone loss at implant-supported crowns with titanium bases [44].

## Conclusion

Within the limitations of this study, the utilization of an allogenic CS with the described protocol did lead to a significant thickening of the STH. However, it did not achieve a level of STH thickening sufficient to significantly reduce MBL compared to the control group. Additionally, patient-related factors such as implant position, history of periodontitis, and smoking status should always be considered as potential risk factors when addressing early peri-implant bone loss.

## Data Availability

No datasets were generated or analysed during the current study.
